# Antioxidant, antiglycation and cytotoxicity evaluation of selected medicinal plants of the Mascarene Islands

**DOI:** 10.1186/1472-6882-12-165

**Published:** 2012-09-29

**Authors:** Fawzi M Mahomoodally, Anwar H Subratty, Ameenah Gurib-Fakim, Muhammad Iqbal Choudhary

**Affiliations:** 1 Department of Health Sciences, Faculty of Science, University of Mauritius, Réduit, 230, Mauritius; 2 Department of Chemistry, Faculty of Science, University of Mauritius, Réduit, Mauritius; 3 Center for Phytotherapy Research, Cyber Tower 2, Ebéne, Mauritius; 4 H.E.J. Research Institute of Chemistry, International Center for Chemical and Biological Sciences, University of Karachi, Karachi, Pakistan

## Abstract

**Background:**

Many indigenous plants of Mascarene Islands have been used in folkloric medicine to manage diabetes but few species have received scientific attention. Selected traditional medicinal plants (*Antidesma madagascariense* Lam. -Euphorbiaceae (AM), *Erythroxylum macrocarpum* O.E.Schulz -Erythroxylaceae (EM), *Pittosporum senacia* Putterl -Pittosporaceae (PS), *Faujasiopsis flexuosa* Lam. C.Jeffrey -Asteraceae (FF), *Momordica charantia* Linn -Cucurbitaceae (MC) and *Ocimum tenuiflorum* L -Lamiaceae (OT) were evaluated for their antioxidant, antiglycation and cytotoxic potential *in vitro*.

**Methods:**

Graded concentrations (1.25-100 μg/mL) of the crude methanolic and water extracts and fractions (dichloromethane, ethyl-acetate, *n*-butanol and water) were evaluated for abilities to scavenge 2,2-diphenyl-2-picrylhydrazyl hydrate (DPPH), nitric oxide (NO), superoxide (SO) radicals and to inhibit lipoxygenase and formation of advanced glycation endproduct (AGE) *in vitro*. The MTT (3-(4, 5-dimethylthazol-2-yl)-2,5-diphenyl tetrazonium bromide) cytotoxicity test was performed on 3T3 cell line.

**Results:**

Only IC_50_ for DPPH, SO, NO and lipoxygenase for AM, FF and OT crude extracts and fractions were comparable to ascorbic acid and quercetin activity. Crude aqueous extracts of AM and FF showed IC_50_ of 4.08 and 3.89 μg/mL respectively for lipoxygenase which was significantly lower (p < 0.05) than quercetin (10.86 ± 0.68 μg/mL). The three crude aqueous extracts of these plants and their *n*-butanol fractions also showed antiglycation activities (p < 0.05) comparable to aminoguanidine. Increasing concentrations (250-2000 μg/mL) of the six crude extracts (Methanol and water) and their fractions did not inhibit mitochondrial respiration as measured by MTT cytotoxicity assay.

**Conclusion:**

AM, FF and OT crude extracts and fractions have potent antioxidant and antiglycation properties with no apparent cytotoxicity and might have prophylactic and therapeutic potentials in the management of diabetes and related complications. Our study tends to validate the traditional use of these medicinal herbs and food plants as complementary and alternative medicines.

## Background

The local population of the Mascarene Islands has a deep-rooted interest in the use of herbal medicine. Many indigenous, endemic and exotic plant species have been used in folkloric medicine to treat and manage various ailments of man including chronic pathologies such as diabetes mellitus (DM) and cardiovascular diseases. Available reports tend to highlight that indigenous folk-medicinal plant preservation and study is vital because such plants are fully adapted to local environment and conditions compared to exotic species [1,2]. Pharmacologically active compounds and phytochemicals isolated from endemic and indigenous plants used in folk medicine have been the areas of interest [3-6]. Currently, several kinds of preparations from various exotic, endemic and indigenous plants are sold as decoctions or “tisanes” in several markets to treat minor ailments and major pathologies, including DM. DM in Mauritius is becoming a devastating scourge with more than 120,000 cases reported and 4.6% death from DM complications such as nephropathy and neuropathy [7]. Moreover, Mauritius has one of the highest prevalence of diabetes in the world with nearly one in five of adults above the age of 30 years suffering from diabetes. Complications and side effects associated with DM and failure of existing antidiabetic drugs are forcing researchers to come up with long term and sustainable solutions for management of diabetes.

Nonetheless, few antidiabetic medicinal plants of the Mascarene Islands particularly Mauritius, have received scientific attention and complete evidence based validation is missing. In the present study, selected endemic and exotic medicinal plants, of the Mascarene Islands, which are traditionally used for management of diabetes, have been investigated for their possible antioxidant, antiglycation and cytotoxicity properties *in vitro*.

## Materials and methods

### Plant materials

Leaves of the endemic and indigenous plants; *Antidesma madagascariense* Lam. - Euphorbiaceae (AM- FM012005), *Erythroxylum macrocarpum* O.E.Schulz - Erythroxylaceae (EM- FM022005), *Pittosporum senacia* Putterl - Pittosporaceae (PS- FM032005), *Faujasiopsis flexuosa* (Lam) C.Jeffrey- Asteraceae (FF- FM042005), common to the Mascarene Islands were collected from Conservation Management Areas (Maccabé forest) situated in the upper humid region of Petrin and Forest-Side conservation area, Mauritius. Exotic plant *Ocimum tenuiflorum* L- Lamiaceae (OT- FM052005) were obtained from the University of Mauritius farm. Fresh *Momordica charantia* Linn- Cucurbitaceae (MC- FM062005) were obtained from a commercial source and prepared as described previously. The Curator of the National Herbarium, at the Mauritius Sugar Industry Research Institute confirmed the identity of the plants and voucher specimen deposited therein. The plant materials were prepared as reported previously [4,5].

### Extract preparation

Powdered (10 g) plant materials were extracted to exhaustion with 50 mL of water in a Soxhlet apparatus for 5 h. The solvent was then distilled off under reduced pressure and temperature (40°C) to afford crude plant extract. The extracts were concentrated *in vacuo* using a rotary evaporator (Model Buchi rotavapor R-114, Switzerland). The resultant concentrate was measured and the gummy material collected in the appropriate solvent for examination. Methanolic extracts were obtained by triple soaking in 80% methanol at room temperature for 3 days and the solvent removed under reduced pressure. The paste-like suspension was diluted in DMSO for further experiments. Crude methanolic extracts were then fractionated by solvent-solvent extraction procedure into dichloromethane, ethyl-acetate, *n*-butanol and aqueous fractions for two successive 24-h periods respectively [3,4,8]. In all, six different extracts and fractions per medicinal plant were tested for biological activity *in vitro*.

### DPPH (2,2-diphenyl-2-picrylhydrazyl hydrate) radical scavenging assay

Radical scavenging activity of the different plant extracts and respective fractions against stable DPPH (2,2-diphenyl-2-picrylhydrazyl hydrate, Sigma-Aldrich) was determined spectrophotometrically [9]. Extract solutions were prepared by dissolving 1 mg/mL of the crude extract in 1 mL of DMSO. The concentration of DPPH was maintained at 300 mM with variable concentrations of sample. The solution of DPPH in ethanol was prepared on a daily basis, and prior to UV measurements. 10 μL (concentration ranged from 12.5 to 10 μg/mL) of each sample was dissolved in DMSO and mixed with 95 mL of DPPH in ethanol. The mixture was dispersed in a 96-well plate and incubated at 37°C for 30 min and the absorbance at 515 nm was measured by 96-well microtitre plate reader (Spectramax plus 384 Molecular Device, USA), and percentage radical scavenging activity was determined in comparison with the DMSO-treated (negative control). Ascorbic acid was used as a positive control. Results were expressed as percentages compared to control and the corresponding IC_50_ calculated.

### Nitric oxide radical inhibition assay

The reaction mixture (100 μL) containing sodium nitroprusside (10 mM, 70 μL), phosphate buffer saline (1 mM, 20 μL) and extract or standard solution (0.1 mL) was incubated at 25°C for 90 min in 96-well microtitre plates. The absorbance of these solutions was measured at 570 nm against the corresponding blank solutions using spectrophotometer (Spectronic Genesys 8, Rochester, USA). Results were expressed as percentages compared to negative control and the corresponding IC_50_ calculated [9]. Ascorbic acid was used as a positive control.

### Superoxide anion radical scavenging activity

In this experiment, the superoxide radicals were generated in 200 μL of phosphate buffer (0.1 M, pH 7.5) containing 40 μL of NBT (80 mM) solution, 40 μL NADH (280 mM) solution and sample solution of graded concentrations (6.25-1000 μg/mL) in water. 10 μL of test samples were dissolved in DMSO and the reaction was started by adding 20 μL of phenazine methosulphate solution (8 mM) to the mixture. The reaction mixture was incubated at 25^o^C for 5 min and the absorbance at 560 nm was measured against the blank at 23^o^C [10] in 96-well microtitre plate reader. Ascorbic acid was used as a positive control and DMSO as negative control. Results were expressed as percentages compared to control and the corresponding IC_50_ calculated.

### Non-enzymatic antiglycation activities

Albumin-derived advanced glycation endproducts (AGEs) were measured using fluorometry as described previously [11,12]. Briefly, 1mg/ml of fatty acid-free bovine serum albumin (BSA) was incubated with D-glucose (200-400 mM) ± 100 μl of herb or spice extracts in 0.2 M potassium phosphate buffered saline (pH 7.4 containing 0.01% sodium azide) at 37°C for defined time periods. Aliquots of the reaction mixture were removed at weekly intervals and fluorescent AGEs were assessed by their emission at 440 nm following excitation at 370nm using a spectrofluorimeter (F-7000 FL) [11]. The reaction was stopped by adding 10 μl of 100% (w/v) tricholoroacetic acid and after ten minutes the mixtures were centrifuged at 10000 g. The precipitate was re-dissolved in alkaline phosphate buffered saline (PBS) and quantified for the relative amount of fluorescent AGEs. Complete inhibition of fluorescent AGEs was assumed to occur when fluorescence was inhibited to that of albumin in the absence of glucose; which was used as a negative control. Any sample giving fluorescence equal to the fluorescence of BSA/glucose implied that there was no inhibition of glycation; whereas, any sample giving fluorescence lower than that of BSA/glucose indicated that there was inhibition of glycation by the extract present. Aminoguanidine was included as a positive control. The percentage inhibition of glycation of each of the negative control and plant extract was calculated as follows: [(Fluorescence of specimen – fluorescence of BSA/Glucose)/ Fluorescence of BSA/Glucose] X 100. Results were expressed as percentages compared to negative control and the corresponding IC_50_ calculated.

### Assay for inhibition of 15-lipoxygenase

Inhibition of 15-lipoxygenase was carried out using soybean lipoxygenase type 1-B (Sigma, St. Louis, MO, USA and/or Fluka, Buchs, Switzerland) as described by Lyckander & Malterud, [13]. Measurements of increase in absorbance at 234 nm for 30-90 sec after enzyme addition were done in 0.2 M borate buffer (pH 7.5) with linoleic acid (134 μM) as substrate and an enzyme concentration of 167 U/mL, using test substance solutions in DMSO or water or (for blanks) DMSO or water alone. Enzyme inhibitory activities were calculated from the values for absorption increase per time unit, as supplied from the software of the spectrometer. Three or more parallels for blanks and for samples were measured [13,14]. Results were expressed as percentages compared to negative control and the corresponding IC_50_ calculated.

### MTT (3-(4, 5-dimethylthazol-2-yl)-2, 5-diphenyl tetrazonium bromide) cytotoxicity assay

In the present investigation, 3T3 cell line clone A31 adherent cell line was used and cytotoxicity on these cells was assessed as described previously [15,16]. For each experiment, cultures were seeded from frozen stocks. 3T3 cells were maintained in Ham’s F12 medium supplemented with 10% fetal calf serum (FBS) and 1% antibiotic solution. All cell lines were incubated at 37°C in a 5% CO_2_ atmosphere and were in the logarithmic phase of growth at the time of the neutral red (NR) and tetrazolium (MTT) assays. Cells were harvested and seeded into 96-well tissue culture plates at a density of 1x10^4^ cells per well of aliquots of medium (200 μl). The cells were allowed to adhere to the wells for 24 h at 37°C in a humidified atmosphere optimized with 5% CO_2_ in air. The next day, the plant extract/fractions were added at the desired final concentrations and incubated for 72h. All experiments were performed at least four times. Phosphate-buffered saline (PBS) was used as a negative. After the 72 h exposure period, the toxic endpoints were determined at 570 and 620 nm. Viability was defined as the ratio (expressed as a percentage) of absorbance of treated cells to untreated cells that served as negative control.

### Statistical analysis

All data were expressed as means ± SD for three experiments. The difference between the means ± SD of the antioxidant and antiglycation activity between the control (without plant extract) and experimental group were assessed using the one way ANOVA test. P values < 0.05 were considered statistically significant. Statistical analyses were performed using Excel software (Microsoft 2007) and SPSS version 16.0 for Windows 2007 [5,6].

## Results

### Antioxidant activities

Antioxidant properties of the crude extracts and different fractions of the six plants were evaluated for their abilities to scavenge 2,2-diphenyl-2-picrylhydrazyl hydrate (DPPH), nitric oxide (NO) and superoxide anion (SO) radicals *in vitro*. The results obtained are summarized in Tables 1, 2, 3. Graded concentrations of the different extracts and fractions tested ranged from 12.5-100 μg/mL and the results expressed as percentage radical scavenging activities. As shown in Tables 1, 2, 3 only crude methanol and water extracts and corresponding fractions of AM, FF and OT were found to posses significant radical scavenging abilities (though moderate) compared to the control (without extract added).

**Table 1:** DPPH radical scavenging activity of crude extracts and fractions of 6 plants from the Mascarene Islands

**Concentrations** (**μg**/**mL**)	**DPPH scavenging activity**					
	**Crude water extract**	**Crude methanol extract**	**Dichloromethane**	**Ethylacetate**	***n***-**butanol**	**Water fraction**
100	9.23 ± 2.29	8.63 ± 1.03	4.26 ± 0.69	13.26 ± 0.96	14.59 ± 1.09	11.23 ± 1.01
	(20.13 ± 3.46*)	(23.39 ± 1.36)	(87.52 ± 6.59*)	(92.86 ± 7.16*)	(93.68 ± 8.69*)	(80.13 ± 7.54*)
	{67.46 ± 9.56*}	{75.56 ± 7.65*}	{78.67 ± 6.15*}	{93.45 ± 9.14*}	{97.08 ± 11.23*}	{72.78 ± 5.36*}
	[90.14 ± 10.07*]	[91.56 ± 10.69*]	[67.59 ± 8.09*]	[90.13 ± 6.69*]	[94.86 ± 8.09*]	[88.49 ± 7.23*]
	∫5.36 ± 0.99∫	∫11.29 ± 1.05∫	∫9.86 ± 1.11∫	∫7.42 ± 0.39∫	∫11.36 ± 1.00∫	∫8.59 ± 1.96∫
	∣9.86 ± 1.23∣	∣12.03 ± 1.06∣	∣18.16 ± 2.13∣	∣15.69 ± 1.39∣	∣15.46 ± 0.23∣	∣7.39 ± 1.09∣
50	4.21 ± 1.23	3.98 ± 1.11	2.04 ± 0.23	7.49 ± 0.96	9.18 ± 0.23	5.13 ± 1.03
	(14.36 ± 0.89)	(12.39 ± 0.69)	(61.43 ± 5.67*)	(75.67 ± 4.56*)	(75.64 ± 3.68*)	(62.14 ± 5.67*)
	{52.16 ± 7.65*}	{58.89 ± 5.94*}	{63.19 ± 7.62*}	{72.14 ± 8.56*}	{76.37 ± 9.56*}	{55.09 ± 5.53*}
	[69.64 ± 7.19*]	[64.59 ± 6.36*]	[41.68 ± 8.09*]	[53.67 ± 9.36*]	[71.69 ± 7.36*]	[62.13 ± 5.36*]
	∫1.23 ± 0.16∫	∫7.09 ± 0.63∫	∫3.67 ± 0.26∫	∫4.09 ± 0.19∫	∫5.13 ± 0.36∫	∫5.29 ± 1.22∫
	∣4.61 ± 0.96∣	∣7.01 ± 0.69∣	∣10.69 ± 1.29∣	∣8. 69 ± 0.36∣	∣8.63 ± 0.19∣	∣3.59 ± 0.67∣
25	2.15 ± 0.60	1.23 ± 0.22	1.09 ± 0.09	5.29 ± 0.63	5.69 ± 0.11	2.39 ± 0.36
	(9.19 ± 1.13)	(8.46 ± 1.03)	(46.49 ± 1.98*)	(53.36 ± 3.79*)	(62.14 ± 1.69*)	(50.23 ± 2.97*)
	{40.14 ± 6.15*}	{42.15 ± 6.54*}	{40.18 ± 6.81*}	{57.09 ± 6.36*}	{54.76 ± 3.39*}	{48.13 ± 4.46*}
	[42.18 ± 8.39*]	[45. 67 ± 5.01*]	[22.19 ± 5.13*]	[32.19 ± 5.12*]	[51.63 ± 3. 16*]	[47.16 ± 4.16*]
	∫0.69 ± 0.01∫	∫4.26 ± 0.69∫	∫1.14 ± 0.08∫	∫2.07 ± 0.69∫	∫2.19 ± 0.12∫	∫3.19 ± 0.18∫
	∣2.19 ± 0.13∣	∣3.69 ± 0.23∣	∣5.18 ± 0.96∣	∣5.19 ± 0.08∣	∣3.14 ± 0.08∣	∣1.56 ± 0.16∣
12.5	1.26 ± 0.35	0.68 ± 0.13	0.97 ± 0.11	2.09 ± 0.16	2.57 ± 0.09	0.98 ± 0.15
	(4.16 ± 0.56)	5.34 ± 0.87	(30.69 ± 1.63*)	(38.56 ± 2.67*)	(41.64 ± 1.59*)	(28.26 ± 2.39*)
	{29.46 ± 3.87}*	{34.19 ± 5.87*}	{32.49 ± 4.57*}	{40.18 ± 5.42*}	{36.15 ± 2.23*}	{29.86 ± 5.21*}
	[28.49 ± 1.69*]	[29.19 ± 1.23*]	[12.19 ± 0.99]	[24.19 ± 1.08*]	[31.46 ± 1.53*]	[29.18 ± 1.39*]
	∫0.56 ± 0.01∫	∫2.13 ± 0.23∫	∫0.59 ± 0.01∫	∫0.11 ± 0.01∫	∫1.39 ± 0.03∫	∫1.09 ± 0.01∫
	∣1.01 ± 0.01∣	∣1.69 ± 0.02∣	∣2.09 ± 0.39∣	∣1.69 ± 0.01∣	∣0.69 ± 0.01∣	∣1.06 ± 0.01∣

*Note*. Values are means ± SD of three independent experiments. EM (AM) {FF} [OT] ∫PS∫ ∣MC∣. *Values significantly different (p < 0.05) from the control in each group.

**Table 2:** Superoxide radical scavenging activity of crude extracts and fractions of 6 plants from the Mascarene Islands

**Concentrations** (**μg**/**mL**)	**Superoxide scavenging activity**					
	**Crude water extract**	**Crude methanol extract**	**Dichloromethane**	**Ethylacetate**	***n***-**butanol**	**Water fraction**
100	5.23 ± 1.23	2.23 ± 0.69	10.89 ± 1.46	19.36 ± 2.30*	12.69 ± 1.34	2.39 ± 0.36
	(82.39 ± 7.34*)	(98.97 ± 5.37*)	(47.77 ± 6.34*)	(99.53 ± 7.53*)	(98.67 ± 5.04*)	(68.07 ± 4.31*)
	{65.68 ± 9.67*}	{74.23 ± 7.68*}	{22.69 ± 1.09*}	{55.44 ± 2.39*}	{82.80 ± 12.69*}	{86.59 ± 10.18*}
	[40.69 ± 6.67*]	[36.56 ± 3.79*]	[21.26 ± 1.69*]	[80.69 ± 10.69*]	[72.39 ± 9.68*]	[40.35 ± 3.69*]
	∫2.03 ± 1.03∫	1.03 ± 0.23∫	∫5.36 ± 0.96∫	∫15.69 ± 1.69*∫	∫5.67 ± 0.67∫	∫8.67 ± 1.23∫
	∣7.29 ± 1.06∣	∣5.09 ± 1.00∣	∣12.29 ± 1.27∣	∣32.93 ± 2.67*∣	∣10.24 ± 2.13∣	∣8.23 ± 0.96∣
	2.56 ± 0.99	1.26 ± 0.11	7.79 ± 1.47	9.67 ± 1.67	6.35 ± 0.69	1.09 ± 0.37
50	(58.64 ± 3.15*)	(70.64 ± 5.43*)	(23.67 ± 1.08*)	(74.59 ± 6.07*)	(72.19 ± 3.32*)	(52.39 ± 2.01*)
	{40.29 ± 6.54*}	{57.19 ± 6.45*}	{11.09 ± 0.98}	{30.27 ± 2.79*}	{52.69 ± 9.67*}	{50.19 ± 5.67*}
	[23.68 ± 4.56*]	[13.12 ± 2.19]	[12.18 ± 2.09]	[52.39 ± 6.59*]	[47.69 ± 6.56*]	[26.67 ± 2.01*]
	∫1.09 ± 0.03∫	∫0.96 ± 0.01∫	∫1.29 ± 0.67∫	∫7.16 ± 0.84∫	∫3.67 ± 0.38∫	∫5.67 ± 0.96∫
	∣3.19 ± 1.01∣	∣2.39 ± 0.16∣	∣9.25 ± 0.67∣	∣18.39 ± 1.97∣	∣7.24 ± 1.01∣	∣6.58 ± 0.64∣
	0.56 ± 0.01	0.59 ± 0.01	4.69 ± 1.06	5.49 ± 1.39	4.56 ± 0.34	0.67 ± 0.09
25	(41.19 ± 2.04*)	(56.38 ± 2.38*)	(11.29 ± 0.67)	(59.78 ± 3.08*)	(54.39 ± 1.17*)	(35.56 ± 1.86*)
	{18.09 ± 2.31*}	{41.09 ± 3.47*}	{6.57 ± 0.73}	{14.67 ± 1.98}	{36.48 ± 5.24*}	{28.49 ± 1.38*}
	[14.29 ± 3.97*]	[9.68 ± 1.09]	[6.79 ± 1.61]	[38.19 ± 6.64*]	[29.36 ± 2.17*]	[13.19 ± 1.39]
	∫0.22 ± 0.01∫	∫0.56 ± 0.02∫	∫0.56 ± 0.24∫	∫4.56 ± 0.99∫	∫1.29 ± 0.35∫	∫4.96 ± 0.37∫
	∣1.29 ± 1.00∣	∣1.18 ± 0.23∣	∣6.58 ± 2.39∣	∣10.67 ± 2.01∣	∣4.12 ± 0.96∣	∣3.28 ± 0.28∣
	0.06 ± 0.01	0.13 ± 0.01	2.65 ± 0.67	2.67 ± 0.69	1.67 ± 0.09	0.01± 0.01
12.5	(28.98 ± 1.31*)	(42.46 ± 2.28*)	(7.69 ± 0.17)	(42.19 ± 1.38*)	36.49 ± 0.37*	(17.19 ± 0.67*)
	{10.39 ± 0.96}	{17.19 ± 1.96*}	{2.39 ± 0.24}	{7.09 ± 0.97}	{15.36 ± 1.74*}	{15.14 ± 2.67*}
	[17.69 ± 1.46*]	[4.68 ± 0.69]	[4.67 ± 1.09]	[13.18 ± 1.29]	[13.17 ± 0.96]	[7.71± 1.09]
	∫0.13 ± 0.01∫	∫0.00 ± 0.00∫	∫0.25 ± 0.01∫	∫2.01 ± 0.76∫	∫0.27 ± 0.01∫	∫1.23 ± 0.27∫
	∣0.59 ± 0.01∣	∣0.69 ± 0.01∣	∣2.18 ± 0.29∣	∣5.97 ± 1.91∣	∣1.69 ± 0.86∣	∣1.28 ± 0.11∣

*Note*. Values are means ± SD of three independent experiments. EM (AM) {FF} [OT] ∫PS∫ ∣MC∣. *Values significantly different (p < 0.05) from the control in each group.

**Table 3:** Nitric oxide radical scavenging activity of crude extracts and fractions of 6 plants from the Mascarene Islands

**Concentrations** (**μg**/**mL**)	**Nitric oxide scavenging activity**					
	**Crude water extract**	**Crude methanol extract**	**Dichloromethane**	**Ethylacetate**	***n***-**butanol**	**Water fraction**
100	1.23 ± 0.12	5.36 ± 0.94	3.46 ± 0.11	0.96 ± 0.09	7.68 ± 1.01	10.67 ± 1.37
	(59.39 ± 2.39*)	(62.73 ± 4.67*)	(33.26 ± 2.54*)	(65.02 ± 6.49*)	(65.56 ± 7.56*)	(45.21 ± 2.98*)
	{59.63 ± 3.06*}	{61.46 ± 2.39*}	{54.97 ± 3.01*}	{72.39 ± 3.36 *}	{56.51 ± 4.08*}	{56.04 ± 2.86*}
	[44.36 ± 3.56*]	[47.78 ± 3.07*]	[52.72 ± 3.24*]	[48.97 ± 2.01*]	[52.69 ± 4.01*]	[55.24 ± 3.34*]
	∫4.36 ± 0.68∫	∫7.46 ± 1.01∫	∫10.37 ± 1.39∫	∫11.09 ± 1.09 ∫	∫9.46 ± 1.13 ∫	∫5.37 ± 0.98∫
	∣3.64 ± 0.18*∣	∣5.69 ± 0.54∣	∣31.34 ± 2.07*∣	∣22.36 ± 1.01*∣	∣28.69 ± 0.68*∣	∣5.78 ± 0.67∣
	0.56 ± 0.03	2.69 ± 0.64	1.69 ± 0.05	0.23 ± 0.07	6.49 ± 0.96	7.39 ± 0.96
50	(38.64 ± 1.69*)	(41.31 ± 3.29*)	(15.36 ± 1.06)	(43.69 ± 5.46)	(41.29 ± 3.69*)	(21.68 ± 1.07*)
	{32.46 ± 3.67*}	{40.16 ± 1.07*}	{27.68 ± 1.07*}	{42.09 ± 2.67*}	{31.10 ± 1.37*}	{29.76 ± 2.01*}
	[29.34 ± 2.31*]	[23.67 ± 2.01*]	[30.37 ± 1.69*]	[27.19 ± 1.06*]	[28.67 ± 1.06*]	[30.19 ± 1.67*]
	∫2.39 ± 0.59∫	∫6.37 ± 1.00∫	∫6.54 ± 0.97∫	∫6.31 ± 1.01 ∫	∫3.31 ± 0.67∫	∫4.67 ± 0.64∫
	∣1.09 ± 0.0∣	∣2.36 ± 0.34∣	∣28.37 ± 1.37∣	∣9.37 ± 0.96∣	∣26.34 ± 1.01∣	∣3.67 ± 0.97∣
25	0.18 ± 0.02	0.96 ± 0.01	0.97 ± 0.03	0.19 ± 0.01	2.69 ± 0.31	4.19 ± 0.43
	(22.67 ± 3.67*)	(28.46 ± 2.87)	(7.09 ± 0.96)	(26.59 ± 1.09*)	(29.64 ± 2.98*)	(11.37 ± 0.87)
	{17.19 ± 1.64*}	{22.06 ± 1.11*}	{12.08 ± 0.67}	{28.19 ± 1.37*}	{9.65 ± 0.79}	{10.09 ± 1.04}
	[19.17 ± 1.08*]	[10.19 ± 1.36]	[11.37 ± 0.67]	[13.19 ± 0.37]	[10.36 ± 1.01]	[27.19 ± 1.06*]
	∫0.36 ± 0.37∫	∫4.09 ± 0.64∫	∫4.37 ± 0.36∫	∫3.97 ± 0.63∫	∫1.34 ± 0.52 ∫	∫3.17 ± 0.52∫
	∣0.19 ± 0.01∣	∣1.94 ± 0.19∣	∣9.37 ± 1.07∣	∣6.54 ± 0.67∣	∣11.97 ± 0.67∣	∣1.67 ± 0.61∣
12.5	0.09 ± 0.01	0.23 ± 0.01	0.15 ± 0.01	0.06 ± 0.01	0.37 ± 0.01	2.64 ± 0.19
	(12.19 ± 1.15)	(19.29 ± 1.39*)	(1.23 ± 0.13)	(10.69 ± 0.69)	(18.67 ± 1.08*)	(5.36 ± 0.36)
	{9.86 ± 0.97}	{10.36 ± 0.67}	{4.89 ± 0.55}	{12.67 ± 0.68}	{2.98 ± 0.32}	{6.17 ± 0.63}
	[9.68 ± 1.23]	[6.45 ± 0.37]	[4.67 ± 0.23]	[7.09 ± 0.22]	[6.98 ± 0.67]	[11.09 ± 0.37]
	∫0.09 ± 0.02∫	∫2.19 ± 0.34∫	∫1.09 ± 0.23∫	∫0.67 ± 0.23∫	∫0.67 ± 0.03∫	∫0.96 ± 0.09∫
	∣0.00 ± 0.00∣	∣0.37 ± 0.09∣	∣2.39 ± 0.23∣	∣ 3.67 ± 0.54∣	∣ 6.37 ± 0.37∣	∣0.98 ± 0.09∣

*Note*. Values are means ± SD of three independent experiments. EM (AM) {FF} [OT] ∫PS∫ ∣MC∣. *Values significantly different (p < 0.05) from the control in each group.

The percentage DPPH radical scavenging activities of AM, FF and OT ranged between 20.13 ± 3.46 to 93.68 ± 8.69, 29.46 ± 3.87 to 97.08 ± 11.23, and 24.19 ± 1.08 to 94.86 ± 8.09 respectively. For AM, the crude water and methanol extracts showed poor activities, unlike FF and OT whereby all the extracts and fractions were active.

The percentage SO radical scavenging of AM, FF and OT ranged between 17.19 ± 0.67 to 99.53 ± 7.53, 15.14 ± 2.67 to 86.59 ± 10.18, and 14.29 ± 3.97 to 80.69 ± 10.69 respectively. For both AM and OT, the highest scavenging activities were observed for the ethylacetate fraction 99.53 ± 7.53 and 80.69 ± 10.69 respectively. A fair activity was observed for PS and MC ethylacetate fractions (15.69 ± 1.69 and 32.93 ± 2.67 respectively).

The percentage NO radical scavenging of AM, FF and OT ranged between 65.56 ± 7.56 to 19.29 ± 1.39, 17.19 ± 1.64 to 72.39 ± 3.36, and 19.17 ± 1.08 to 55.24 ± 3.34 respectively. The highest activities for AM, FF and OT were recorded for *n*-Butanol (65.56 ± 7.56), ethylacetate (72.39 ± 3.36) and water fraction (55.24 ± 3.34) respectively.

**Figure 1:**
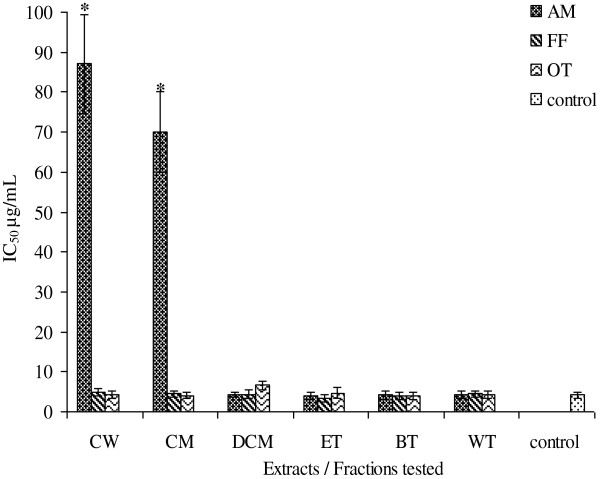
**IC_50 _for DPPH radical scavenging properties of AM**, **FF and OT crude extracts and fractions**.

**Figure 2:**
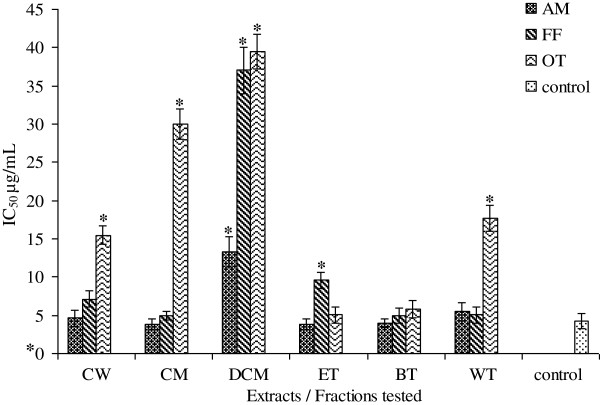
**IC_50 _for SO radical scavenging properties of AM**, **FF and OT crude extracts and fractions**.

**Figure 3:**
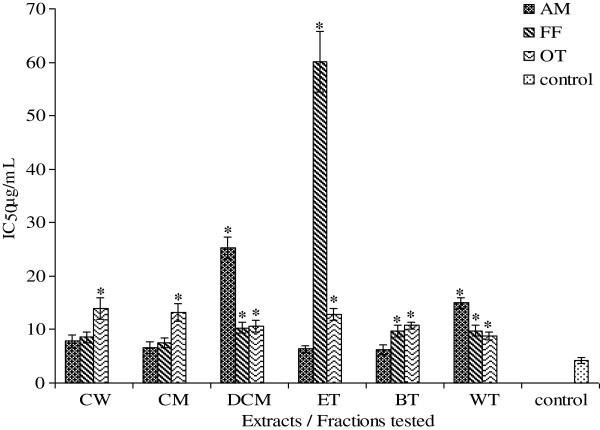
**IC_50 _ for NO radical scavenging properties of AM**, **FF and OT crude extracts and fractions**.

The DPPH IC_50_ values for AM, FF and OT (Figure 1) ranged from 3.94 to 87.05 μg/ mL, 3.39 to 4.87 μg/mL, and 4.00 to 6.67 μg/mL respectively. The SO IC_50_ values for AM, FF and OT (Figure 2) ranged from 3.81 to 13.26 μg/mL, 4.95 to 37.01 μg/mL, and 5.05 to 39.48 μg/mL. The NO IC_50_ values for AM, FF and OT (Figure 3) ranged from 6.29 to 25.24 μg/mL, 6.06 to 10.30 μg/mL, and 8.83 to 13.95 μg/mL. All extracts and fractions showed DPPH IC_50_ values more or less similar to the positive control ascorbic acid 4.16 μg/mL except the crude water and methanol extracts (87.05 and 70.09 μg/mL respectively).The ethylacetate fractions of AM and FF showed best activities (3.94 μg/mL and 3.39 respectively), whereas the *n*-butanol fraction of OT was the most potent extract showing an IC_50_ of 4.00 μg/mL. Only the dichloromethane fraction for AM, FF and OT; and crude water extracts of FF and OT; and crude methanol and water fraction of OT showed SO IC_50_ values higher (p < 0.05) than the positive control ascorbic acid (4.30 μg/mL). The best SO IC_50_ value for AM was the ethylacetate fraction (3.81 μg/mL), for FF crude methanol/water extract (4.95 μg/mL) and OT an IC_50_ of 5.05 μg/mL.

### Antiglycation activities

**Figure 4:**
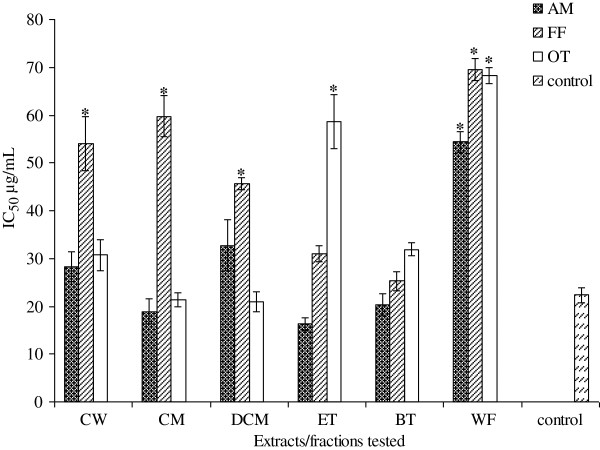
**IC_50 _for antiglycation properties of AM**, **FF and OT crude extracts and fractions**.

The concentrations of extracts and fractions tested for antiglycation activities ranged from 250 to 2000 μg/mL (Table 4). Results showed that AM, FF, OT and MC possessed antiglycation activities compared to the control experiment (without the extracts added) and comparable to the anti-glycation drug aminoguanidine. From the results obtained, it was observed that the degree of antiglycation activities varies considerably from plant to plant and also on the different fractions tested. The ethylacetate fraction of AM showed the highest percentage activity (96.63 ± 10.36) and the least active fraction was the water fraction (62.31 ± 7.65) at 2000 μg/mL. All the tested extracts and fractions showed significant activities (P < 0.05) except the water fraction of AM which showed poor activity. The IC50 values for the active fractions are summarized in Figure 4. As can been seen, the IC50 values ranged from 16.29 ± 1.32 to 54.35 ± 2.12, 25.31 ± 1.98 to 69.54 ± 2.32, and 21.01 ± 2.06 to 68.29 ± 1.68 μg/mL for AM, FF and OT respectively. However, only a few extracts and fractions exhibited inhibitory activities similar to the drug aminoguanidine.

**Table 4:** Anti-glycation activity of crude extracts and fractions of 6 plants from the Mascarene Islands

**Concentrations** (**μg**/**mL**)	**Antiglycation**					
	**Crude water extract**	**Crude methanol extract**	**Dichloromethane**	**Ethylacetate**	***n***-**butanol**	**Water fraction**
2000	75.63 ± 7.89*	86.35 ± 5.65*	78.69 ± 6.54*	96.63 ± 10.36*	84.65 ± 6.35*	62.31 ± 7.65*
	{62.31 ± 6.35*}	{56.31 ± 7.09*}	{68.67 ± 5.32*}	{76.34 ± 5.32*}	{80.36 ± 9.25*}	{55.37 ± 1.32*}
	[78.63 ± 6.54*]	[80.56 ± 8.62*]	[82.63 ± 9.32*]	[56.34 ± 2.36*]	[71.23 ± 7.21*]	[56.31 ± 3.54*]
	(25.36 ± 1.32*)	(32.63 ± 2.32*)	(56.32 ± 2.36*)	(16.32 ± 0.09*)	(37.68 ± 1.84*)	(10.36 ± 0.37)
	∫3.64 ± 0.03∫	∫6.35 ± 0.32∫	∫9.36 ± 0.32∫	∫7.65 ± 0.32∫	∫6.34 ± 0.32∫	∫1.26 ± 0.01∫
	∣6.32 ± 0.21∣	∣7.62 ± 1.21∣	∣6.36 ± 0.02∣	∣5.63 ± 0.21∣	∣6.32 ± 0.02∣	∣1.36 ± 0.06∣
1000	61.34± 5.63*	72.36 ± 5.63*	56.34 ± 5.65*	78.64 ± 6.98*	70.32 ± 2.36*	41.12 ± 3.65*
	{40.65 ± 5.32*}	{34.67 ± 3.21*}	{42.36 ± 3.21*}	{59.37 ± 4.31*}	{64.65 ± 7.69*}	{33.14 ± 1.65*}
	[56.36 ± 7.61*]	[67.66 ± 7.24*]	[72.38 ± 5.62*]	[38.62 ± 1.21*]	[52.36 ± 3.32*]	[36.52 ± 2.87*]
	(10.23 ± 0.32)	(11.23 ± 0.65)	(26.35 ± 0.64*)	(11.06 ± 0.21)	(10.36 ± 0.67)	(7.36 ± 0.06)
	∫1.23 ± 0.12∫	∫2.36 ± 0.64∫	∫4.56 ± 0.65∫	∫ 6.35 ± 0.21∫	∫4.15 ± 0.11∫	∫2.36 ± 1.01∫
	∣5.12 ± 0.21∣	∣2.32 ± 0.64∣	∣5.63 ± 0.03∣	∣3.32 ± 0.21∣	∣5.21 ± 0.63∣	∣2.31 ± 0.21∣
500	42.62 ± 2.65*	56.64 ± 8.98*	28.36 ± 6.68*	59.61 ± 7.65*	54.61 ± 6.36*	30.14 ± 3.65*
	{22.34 ± 5.65*}	{12.36 ± 0.98*}	{26.34 ± 8.63*}	{39.87 ± 2.32*}	{52.31 ± 5.32*}	{19.69 ± 2.32*}
	[32.16 ± 3.21*]	[52.34 ± 2.36*]	[52.31 ± 3.61*]	[11.23 ± 0.04]	[46.35 ± 5.21*]	[28.61 ± 3.54*]
	(7.23 ± 0.65)	(6.32 ± 1.21)	(9.36 ± 1.21)	(6.23 ± 0.03)	) 1.3 6.23±)	(2.36 ± 0.11)
	∫0.14 ± 0.03∫	∫0.36 ± 0.06∫	∫2.36 ± 0.57∫	∫2.36 ± 0.09∫	∫3.46 ± 1.09∫	∫1.56 ± 0.61∫
	∣7.36 ± 1.65∣	∣1.23 ± 0.41∣	∣4.24 ± 0.54∣	∣1.11 ± 0.03∣	∣2.35 ± 0.02∣	∣0.21 ± 0.01∣
250	29.65 ± 2.32*	39.65 ± 5.65*	14.23 ± 2.65*	42.31 ± 6.35*	35.64 ± 4.09*	13.29 ± 3.65
	{10.36 ± 2.35}	{6.13 ± 1.35}	{12.67 ± 0.36}	{13.64 ± 1.21}	{16.34 ± 2.31*}	{8.98 ± 1.25}
	[28.96 ± 1.68*]	[39.78 ± 1.91*]	[34.13 ± 2.31*]	[9.63 ± 0.11]	[32.97 ± 1.63*]	[11.23 ± 0.57]
	(2.36 ± 0.09)	(4.35 ± 0.68)	(5.12 ± 0.08)	(2.32 ± 0.01)	(4.12 ± 0.37)	(1.12 ± 0.08)
	∫0.11 ± 0.03∫	∫0.56 ± 0.09∫	∫0.16 ± 0.01∫	∫0.15 ± 0.05∫	∫0.36 ± 0.01∫	∫0.36 ± 0.09∫
	∣0.23 ± 0.17∣	∣0.36 ± 0.09∣	∣2.98 ± 0.06∣	∣0.68 ± 0.05∣	∣0.69 ± 0.03∣	∣0.00 ± 0.00∣

*Note*. Values are means ± SD of three independent experiments. AM {FF}[OT] (MC) ∫EM∫ ∣PS∣. *Values significantly different (p < 0.05) from the control (without extract) in each group.

The ethylacetate, crude methanol and n-butanol fractions of AM were the most active fractions.

Nonetheless, the most potent fraction appears to be *n*-butanol fractions for all the three plants evaluated.

### Lipoxygenase activity

The ability of the six plants crude extracts and their fractions to inhibit the enzyme lipoxygenase is summarized in Table 5. The concentrations of the extracts tested ranged from 1.25 to 10 μg/mL. The extracts and fractions showed widely varying activity. Again, AM, FF and OT were the most active plants, with FF being the most potent plant. However, MC seemed to exhibit a moderate anti-lipoxygenase activity at higher concentration only (10 μg/mL).

**Table 5:** Lipoxygenase activity of crude extracts and fractions of 6 plants from the Mascarene Islands

**Concentrations** (**μg**/**mL**)	**Lipoxygenase inhibition**					
	**Crude water extract**	**Crude methanol extract**	**Dichloromethane**	**Ethylacetate**	***n***-**butanol**	**Water fraction**
10	82.46 ± 10.09*	79.75 ± 10.07*	77.88 ± 6.37*	53.71 ± 4.17*	27.46 ± 1.77*	32.80 ± 1.31*
	{97.06 ± 11.67*}	{98.32 ± 7.64*}	{81.21 ± 5.24*}	{63.94 ± 3.13*}	{74.98 ± 2.23*}	{67.81 ± 4.47*}
	[53.69 ± 2.36*]	[38.88 ± 1.09*]	[52.54 ± 0.95*]	[53.31 ± 1.26*]	[17.87 ± 1.01*]	[48.64 ± 2.07*]
	(13.59 ± 1.01)	(23.03 ± 0.95*)	(41.13 ± 1.68*)	(30.96 ± 1.33*)	(13.35 ± 0.67)	(13.11 ± 0.67)
	∫10.39 ± 0.96∫	∫9.39 ± 1.01∫	∫9.45 ± 0.34∫	∫11.69 ± 0.66∫	∫12.39 ± 1.03∫	∫ 9.68 ± 0.91∫
	∣1.23 ± 0.02 ∣	∣5.62 ± 0.68∣	∣0.96 ± 0.02∣	∣2.36 ± 0.02∣	∣14.96 ± 0.66*∣	∣2.85 ± 0.51∣
5	69.37 ± 9.37*	62.69 ± 6.11*	58.37 ± 3.64*	28.67 ± 3.09*	11.09 ± 0.67	19.67 ± 0.99
	{70.16 ± 5.55* }	{83.67 ± 4.36*}	{68.69 ± 2.01*}	{42.67 ± 3.15*}	{56.04 ± 2.16*}	{46.39 ± 3.37*}
	[37.11 ± 1.39*]	[18.39 ± 2.67*]	[29.67 ± 0.36*]	[36.56 ± 0.76*]	[9.36 ± 0.67]	[32.05 ± 1.16*]
	(7.51 ± 0.77)	(16.37 ± 0.88*)	(28.34 ± 1.09*)	(14.36 ± 1.45*)	(7.57 ± 0.84)	(8.34 ± 0.34)
	∫9.67 ± 0.82∫	∫7.64 ± 0.36∫	∫6.37 ± 0.22∫	∫ 6.37 ± 0.98∫	∫7.72 ± 0.23∫	∫5.37 ± 0.62∫
	∣0.96 ± 0.03∣	∣2.37 ± 0.11∣	∣0.57 ± 0.01∣	∣1.09 ± 0.03∣	∣ 7.09 ± 0.35∣	∣1.06 ± 0.02∣
2.5	56.09 ± 7.67*	50.37 ± 3.07*	32.67 ± 1.06*	20.37 ± 2.01*	7.19 ± 0.65	9.37 ± 0.67
	{53.37 ± 4.31*}	{62.37 ± 3.16*}	{50.37 ± 2.67*}	{37.65 ± 1.11*}	{39.67 ± 1.97*}	{35.67 ± 2.01*}
	[26.09 ± 1.03*]	[10.09 ± 1.37]	[13.06 ± 0.26]	[27.39 ± 1.03*]	[5.36 ± 0.58]	[24.31 ± 1.09*]
	(3.36 ± 0.17)	(8.67 ± 0.36)	(12.04 ± 1.11)	(8.34 ± 0.67)	(5.01 ± 0.11)	(5.06 ± 0.25)
	∫6.37 ± 0.66∫	∫4.09 ± 0.46∫	∫4.36 ± 0.07∫	∫4.09 ± 0.54 ∫	∫4.46 ± 0.33∫	∫3.64 ± 0.36∫
	∣0.67 ± 0.02∣	∣1.06 ± 0.02∣	∣0.36 ± 0.02∣	∣0.67 ± 0.02∣	∣4.53 ± 0.54∣	∣0.97 ± 0.06∣
1.25	32.37 ± 2.07*	29.67 ± 2.01*	28.03 ± 0.45*	11.09 ± 0.37	2.37 ± 0.53	4.09 ± 0.36
	{42.37 ± 2.01*}	{48.69 ± 2.16*}	{32.69 ± 1.97*}	{13.39 ± 0.12}	{26.67 ± 1.37*}	{29.67 ± 1.36*}
	[10.09 ± 0.96]	[6.37 ± 0.65]	[7.63 ± 0.11]	[11.03 ± 0.15]	[3.34 ± 0.27]	[9.03 ± 0.37]
	(1.55 ± 0.01)	(5.34 ± 0.11)	(9.37 ± 0.15)	(5.34 ± 0.60)	(2.09 ± 0.03)	(2.04 ± 0.11)
	∫2.39 ± 0.09∫	∫1.96 ± 0.02∫	∫2.34 ± 0.03∫	∫2.39 ± 0.23∫	∫1.07 ± 0.07∫	∫1.37 ± 0.01∫
	∣0.29 ± 0.01∣	∣0.64 ± 0.01∣	∣0.24 ± 0.01∣	∣0.47 ± 0.01∣	∣2.07 ± 0.16∣	∣0.17 ± 0.01∣

*Note*. Values are means ± SD of three independent experiments. AM {FF}[OT] (MC) ∫EM∫ ∣PS∣. *Values significantly different (p < 0.05) from the control in each group.

The percentage inhibition of AM ranged from 28.03 ± 0.45 to 82.46 ± 10.09, and the best activity was observed for the crude water extract 82.46 ± 10.09 and the least active fraction which gave significant inhibition was the *n*-butanol fraction. For FF, the percentage enzyme inhibition ranged from 26.67 ± 1.37 to 98.32 ± 7.64, and the best activity was noted for crude methanol extract (98.32 ± 7.64 at a concentration of 10 μg/ml). The inhibition of OT for the enzyme ranged from 24.31 ± 1.09 to 53.69 ± 2.36. The best activity was obtained for the crude water extract (53.69 ± 2.36) and the least active fraction which gave significant inhibition was *n*-butanol (24.31 ± 1.09). All extracts and fractions of MC fruits did not inhibit lipoxygenase activity significantly (p < 0.05) except the crude methanol, dichloromethane and the ethylacetate fractions which gave a percentage inhibition of 23.03 ± 0.95, 41.13 ± 1.68 and 30.96 ± 1.33 respectively. However, at lower concentrations 1.25-2.5 μg/mL, the inhibition was not significantly significant (p > 0.05) compared to the control and hence the IC_50_ value was not calculated.

**Figure 5:**
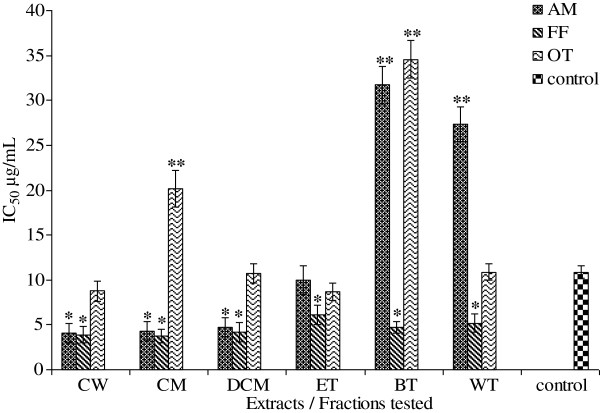
**IC_50 _for lipoxygenase activity of AM**, **FF and OT crude extracts and fractions**.

Figure 5 shows the IC_50_ values for lipoxygenase activity against the tested extracts of the three potent plants (AM, FF and OT). The IC_50_ values for AM, FF and OT ranged from 4.08 to 27.32, 3.89 to 6.13, and 8.70 to 20.14 μg/mL respectively. The best IC_50_ values for AM and FF were obtained for crude water extracts, 4.08 and 3.89 μg/mL respectively, whereas the ethyl acetate fraction of OT was the most active fraction, showing an IC_50_ value of 8.70 μg/mL. The IC_50_ value for quercetin, used as the positive control was 10.86 ± 0.68 μg/mL.

### MTT cytotoxicity test

**Figure 6:**
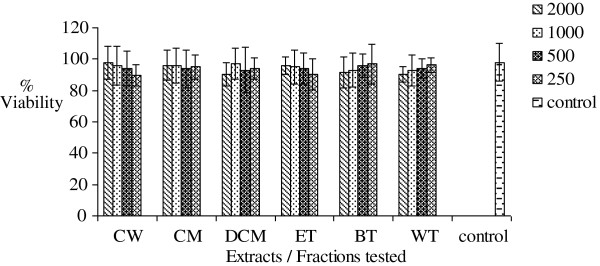
**Effects of *Antidesma madagascariense *on percentage cell viability *in vitro***.

**Figure 7:**
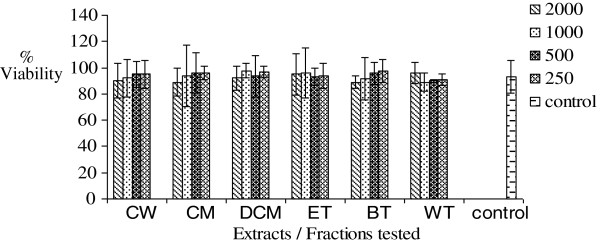
**Effects of *Faujasiopsis flexuosa *on percentage cell viability *in vitro***.

**Figure 8:**
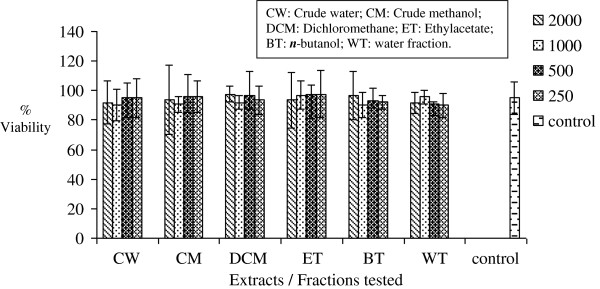
**Effects of *Erythroxylum macrocarpum *on percentage cell viability *in vitro***.

**Figure 9:**
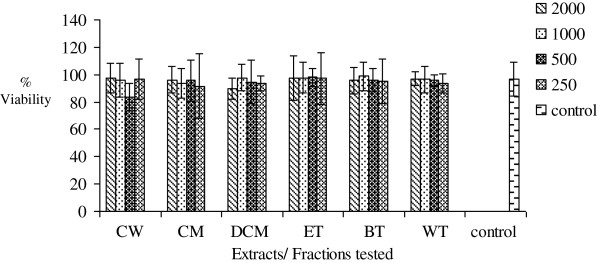
**Effects of PS on percentage cell viability *in vitro***.

**Figure 10:**
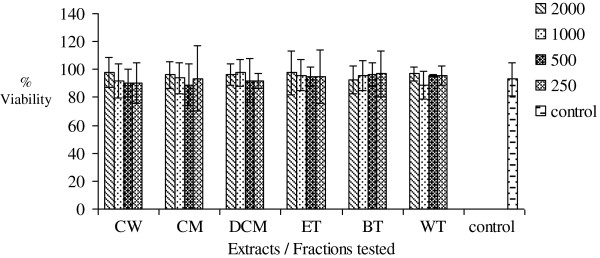
**Effects of *Ocimum tenuiflorum *on percentage cell viability *in vitro***.

**Figure 11:**
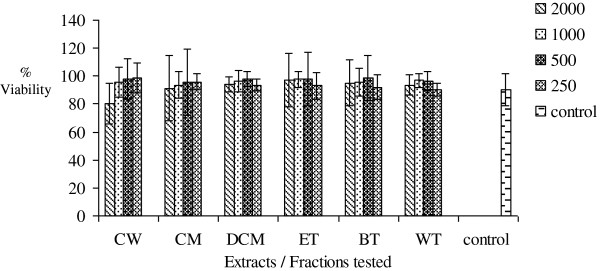
**Effects of *Momordica charantia *on percentage cell viability *in vitro***.

Increasing concentrations (250-2000 μg/mL) of AM, FF, EM, PS, OT and MC crude extracts and fractions were found not to significantly (p >0.05) inhibit mitochondrial respiration as measured by the MTT assay. The results are presented in Figures 6, 7, 8, 9, 10, 11.

## Discussion

Enhanced oxidative stress and the changes in antioxidant capacity, which are observed in both clinical and experimental DM, are thought to contribute to the etiology of chronic diabetic complications [17]. Indeed, an imbalance between formation of reactive oxygen species (ROS) and antioxidants *in vivo* has been suggested to play a major role in multiple chronic diseases including diabetes. ROS, which include free radicals such as superoxide anion radicals, hydroxyl radicals and non free-radical species such as H_2_O_2_ and singlet oxygen, are various forms of activated oxygen. These molecules are exacerbating factors in cellular injury, inflammation, cardiovascular diseases, diabetes and aging process [18]. There have been a growing number of investigations on the potential of antidiabetic medicinal plants as possible antioxidants to prevent or delay chronic diabetic complications. It is generally assumed that frequent consumption of plant derived phytochemicals from vegetables, fruit, tea and medicinal herbs may contribute to the shift in balance toward an adequate antioxidant status.

In the present study, six medicinal plants traditionally used against diabetes were evaluated for their antioxidant properties using standard bioassays such as DPPH, NO and SO radical scavenging potentials. Findings from the present investigation showed that AM, OT and FF were the most active plants for all the three assays as depicted by their low IC_50_ values. However, the degree of inhibition varies considerably and few of the extracts and fractions gave IC_50_ values higher than ascorbic acid, which is a known standard potent antioxidant. Additionally, a clear cut trend in activity for the fractions used was not observed. Nevertheless, the ethylacetate fraction for the three plants tested has been shown to be the most potent extracts. Several reports tend to show that numerous plant derived natural products are effective antioxidants, and many medicinal plants with a long history of use in folk medicine in different countries against a variety of diseases have turned out to be rich sources of antioxidants [19,20]. The advantage of natural antioxidants is their safety and that large oral doses are well tolerated [21]. Many antioxidant compounds, naturally occurring in plant sources have been identified as free radical or active oxygen scavengers. Recently, interest has considerably increased in finding naturally occurring antioxidant for use in foods or medicinal materials to replace synthetic antioxidants, which are being restricted due to their side effects such as carcinogenesis. Natural antioxidants can protect the human body from free radicals and retard the progress of many chronic diseases as well as lipid oxidative rancidity in foods. Hence, studies on natural antioxidants have gained increasingly greater importance [18]. In the investigations reported here, scavenging of the DPPH, SO anion and NO radicals which are commonly used procedures and validated against several other assays for antioxidant activity, including some with relevance for *in vivo* effects [14,22], were employed as radical scavenging assays. For inhibition of an enzymatic peroxidative process, inhibition of 15-lipoxygenase-mediated peroxidation of linoleic acid was used. It is to be noted that, that AM, FF and OT showed scavenging properties for all these *in vitro* assay methods. Lipoxygenase is the enzyme that peroxidizes polyunsaturated fatty acids such as linoleic acid or arachidonic acid to their respective hydro-peroxy derivatives. The six medicinal plants were tested for activity against this enzyme and it was found that AM, FF and OT were the most active fractions, showing good correlation with their antioxidant properties as noted against DDPH, SO and NO. The lipoxygenases have been implicated in a number of pathological states, and 15-lipoxygenase has been suggested to play a role in the development of atherosclerosis and diabetes, probably due to its ability to peroxidize low-density lipoprotein [23,24]. Investigators tend to suggest that an elevated level of lipid peroxides in the plasma of diabetic rats and lipid peroxidation is one of the characteristic features of chronic diabetes. Lipid peroxidation is a free radical induced process leading to oxidative deterioration of polyunsaturated fatty acids and this can be prevented by antioxidants. Additionally, inhibition of soybean 15-lipoxygenase is generally regarded as predictive for inhibition of the mammalian enzyme [25,26]. To this effect, it can be suggested that AM, FF and OT had profound antioxidant properties as supported by the assays carried out in this experiment. However, the phytochemical constituents present in the active extracts and fractions, which are responsible for the observed activity, need to be further investigated. Interestingly, we have recently found the presence of at least tannins, flavonoids and alkaloids in these plants which are known to possess potent antioxidant activity. Hence, the observed antioxidant activity might be due to the presence of any of these constituents or due to a synergistic effect.

A panoply of studies have focused on the factors involved in the pathogenesis of diabetic complications and most seeking effective therapies [27,28]. However, the exact cellular or molecular basis of these complications has not yet been fully elucidated [29]. Hyperglycemia is still considered the principal cause of diabetic complications. Its deleterious effects are attributable, among other things, to the formation of sugar-derived substances called advanced glycation end products (AGEs). AGEs are heterogeneous group of molecules formed from the non-enzymatic reaction of reducing sugars with free amino groups of proteins, lipids, and nucleic acids and their formation is markedly accelerated in diabetes because of the increased availability of glucose [27]. In this study, a simple screening method to measure the inhibitory effects of these extracts and fractions and aminoguanidine on formation of fluorescent AGEs *in vitro* is described. Our system used high concentrations of glucose to speed up the glycation reaction, thus allowing us to undertake the evaluation in an appropriate time-scale as glycation occurs very slowly under physiological conditions [30].

In the present investigation, AM, OT and FF were found to inhibit the formation of AGE *in vitro*. It is suggested that the abilities to inhibit the formation of glycated end products is closely related to the abilities of the antioxidant properties of the plant extracts to scavenge radicals formed during the Maillard reaction which forms the basis of glycation. Interestingly, in the current work, extracts that were found to possess antioxidant properties against lipoxygenase activity were also found to possess antiglycation potential. Furthermore, a few of the extracts were observed to possess antiglycation activities similar to the standard drug aminoguanidine, also known as pimagedine which is a nucleophilic hydrazine compound [29]. Initially, it was thought that aminoguanidine prevented AGE formation by blocking carbonyl groups on Amadori products although it is now known to react with carbonyl groups from reducing sugars [31]. Aminoguanidine, guar gum and acarbose may act as an antioxidant, especially at high concentrations [32]. To this effect, it can be suggested that the antioxidant properties of the AM, FF and OT might to some extent justify the observed antiglycation properties similar to aminoguanidine mode of action.

The MTT assay is a test of metabolic competence based upon assessment of mitochondrial performance relying on the conversion of yellow MTT to the purple formazan derivative by mitochondrial succinate dehydrogenase in viable cells [16]. Increasing concentrations of the six extracts and fractions did not affect mitochondrial respiration as measured by the MTT cytotoxicity assay. Overall, the results of this assay measuring cell integrity showed that these medicinal plants which are widely consumed and used in traditional medicine is not toxic over this concentration range tested.

In this study the crude aqueous extracts were used as per the local tradition of the herbalists. The basis for performing extractions with different solvents was to corroborate and validate the inhibitory activity in the aqueous extractions performed in the traditional manner as well as to search for newer, more potent inhibitory compounds in the organic solvents. To this effect, it was important to assess the antioxidant activities of the crude water extracts in order to validate the medicinal uses of these plants. To this effect, if results from the present study could be extended and translated *in vivo*, then it can be safely suggested that ingestion of crude leaf decoctions of these medicinal plants might be beneficial in the long term management of diabetes. It is believed that these active phytochemicals from the plant extracts could restore the imbalance between formation of ROS and antioxidants observed *in vivo* which has been strongly implicated to play a major role in multiple chronic diseases including diabetes.

## Conclusion

In conclusion, crude aqueous and methanol extracts as well as the organic fractions (mainly ethylacetate and *n*-butanol fractions) of AM, FF and OT only were found to possess potent antioxidant, antiglycation properties and showed no cytotoxicity which tend to support their use in the traditional medicines of the Mascarene Islands. The mechanism of action by which the extracts inhibited glycation is undoubtedly complex. However, it can be argued that the anti-glycation capacity was linked to the antioxidant potentials. Hence, it can be suggested that these plants might have possible prophylactic and therapeutic potentials in the management of diabetes and related complications. Certainly, more studies related to the structure of these phytochemicals and the mechanism(s) of action are required in order to understand their anti-glycation and antioxidant effects.

## Competing interests

No competing financial interests exist.

## Authors’ contributions

FM performed the entire experiment and prepared the manuscript. AGF, IC and AHS participated in the general coordination of the study. All authors read and approved the final manuscript.

## Pre-publication history

The pre-publication history for this paper can be accessed here:

http://www.biomedcentral.com/1472-6882/12/165/prepub

## References

[B1] Qin G Xu R Recent advances on bioactive natural products from Chinese medicinal plants Med Res Rev 1998 18 375 382 10.1002/(SICI)1098-1128(199811)18:6<375::AID-MED2>3.0.CO;2-8 9828038

[B2] Narod F Gurib-Fakim A Subratty AH Phytochemical analysis of selected endemic and indigenous medicinal plants of Mauritius and Rodrigues Res J Chem Env 2004 8 47 50

[B3] Kotwaroo I Mahomoodally MF Gurib-Fakim A Effects of *Arthocarpus heterophyllus* on α-amylase activity *in vitro* Phytother Res 2006 20 228 231 10.1002/ptr.1839 16521114

[B4] Mahomoodally MF Gurib-Fakim A Subratty AH Effects of *Antidesma madagascariense* on the transport of glucose, amino acid, fluid and electrolytes across rat everted intestinal sacs- comparable to insulin action *in vitro* Bri J Biomed Sci 2005 63 1 12 17 10.1080/09674845.2006.11732713 16613135

[B5] Mahomoodally MF Gurib-Fakim A Subratty AH Effect of exogenous ATP on *Momordica charantia* Linn. (Cucurbitaceae) induced inhibition of d-glucose, l-tyrosine and fluid transport across rat everted intestinal sacs *in vitro* J Ethnopharmacol 2007 110 257 263 10.1016/j.jep.2006.09.020 17092672

[B6] Mahomoodally MF Gurib-Fakim A Subratty AH Inhibitory effects of a traditional antidiabetic medicinal fruit extract on the transport of inorganic phosphate and d-glucose across rat everted intestinal sacs- possible relationship with a “Crabtree-effect” J Food Biochem 2012 36 107 115 10.1111/j.1745-4514.2010.00504.x

[B7] Söderberg S Zimmet P Tuomilehto J Increasing prevalence of Type 2 diabetes mellitus in all ethnic groups in Mauritius Diab Med 2005 22 61 68 10.1111/j.1464-5491.2005.01366.x 15606693

[B8] Choudhary MI Begum A Abbaskhan A Phenyl polypropanoids from *Lindelofia stylosa* Chem Pharm Bull 2005 53 11 1469 1471 10.1248/cpb.53.1469 16272735

[B9] Badami S Rai SR Suresh B Antioxidant activity of *Aporosa lindleyana* root J Ethnopharmacol 2005 101 180 184 10.1016/j.jep.2005.04.029 15990259

[B10] Ramachandra MS Venkateswarlu S Subbaraju GV Synthesis of microfolicoumarin, a constituent of *Cedrelopsis microfoliata* Biosci Biotech Biochem 2004 68 9 1995 1997 10.1271/bbb.68.1995 15388980

[B11] Suzuki R Okada Y Okuyama T Two flavone c-glycosides from the style of *Zea mays* with glycation inhibitory activity J Nat Prod 2003 66 564 565 10.1021/np020256d 12713418

[B12] Tang SW Whiteman M Peng NG Characterization of antioxidant and antiglycation properties and isolation of active ingredients from traditional Chinese medicines Free Rad Biol Med 2004 36 12 1575 1587 10.1016/j.freeradbiomed.2004.03.017 15182859

[B13] Lyckander IM Malterud KE Lipophilic flavonoids from *Orthosiphon spicatus* prevent oxidative inactivation of 15-lipoxygenase Prostagland Leuko Essent Fatty Acids 1996 4 4 239 246 10.1016/s0952-3278(96)90054-x 8804120

[B14] Malterub KE Farbrot TL Huse AE Antioxidant and radical scavenging effects of anthraquinones and anthrones Pharmacol 1993 47 S1 77 85 10.1159/000139846 8234446

[B15] Mesaik MA Rahat S Khan KM Synthesis and immunomodulatory properties of selected oxazolone derivatives Bioorg Med Chem 2004 2 9 2049 2057 15080909 10.1016/j.bmc.2004.02.034

[B16] Mesaik MA Zaheer Ul H Murad S Biological and molecular docking studies on coagulin-H: Human IL-2 novel natural inhibitor Mol Immunol 2006 3 11 1855 1163 16375970 10.1016/j.molimm.2005.10.020

[B17] Kang KA Chae S Koh S Protective Effect of *Puerariae* Radix on Oxidative Stress Induced by Hydrogen Peroxide and Streptozotocin Bio Pharm Bull 2005 28 7 1154 1160 10.1248/bpb.28.1154 15997089

[B18] Gulcin I Kufrevioglu OI Oktay M Antioxidant, antimicrobial, antiulcer and analgesic activities of nettle (*Urtica dioica* L.) J Ethnopharmacol 2004 90 2-3 205 215 10.1016/j.jep.2003.09.028 15013182

[B19] Mathisen E Diallo D Andersen M Antioxidants from the bark of *Burkea africana*, an African medicinal plant Phytother Res 2002 16 2 148 153 10.1002/ptr.936 11933117

[B20] Lee H Won NH Kim KH Antioxidant effects of aqueous extract of *Terminalia chebula* in *Vivo* and *in Vitro* Biol Pharm Bull 2005 28 9 1639 1644 10.1248/bpb.28.1639 16141531

[B21] Green K Brand A Murphy M Prevention of mitochondrial oxidative damage as a therapeutic strategy in diabetes Diab 2004 53 S1 S110 S118 10.2337/diabetes.53.2007.s110 14749275

[B22] Tilack JC Banerjee M Mohan H Antioxidant availability of turmeric in relation to its medicinal and culinary uses Phytother Res 2004 10 798 804 10.1002/ptr.1553 15551376

[B23] Steinberg D At last, direct evidence that lipoxygenases play a role in atherogenesis J Clin Invest 1999 103 11 1487 1488 10.1172/JCI7298 10359557 PMC408379

[B24] Cyrus T Pratico D Zhao L Absence of 12/15-lipoxygenase expression decreases lipid peroxidation and atherogenesis in apolipoprotein e-deficient mice Circul 2001 103 18 2277 2282 10.1161/01.CIR.103.18.2277 11342477

[B25] Arambewela LSR Arawwawala LDAM Ratnasooriya WD Antidiabetic activities of aqueous and ethanolic extracts of *Piper beetle* leaves in rats J Ethnopharmacol 2005 102 239 245 10.1016/j.jep.2005.06.016 16055288

[B26] Lapenna D Ciofani G Pierdomenico SD Dihydrolipoic acid inhibits 15-lipoxygenase-dependent lipid peroxidation Free Rad Biol Med 2003 35 10 1203 1209 10.1016/S0891-5849(03)00508-2 14607519

[B27] Peppa M Uribarri J Vlassara H Glucose, advanced glycation end products, and diabetes complications: What is new and what works Clin Diab 2003 21 4 186 187 10.2337/diaclin.21.4.186

[B28] Yamagishi S Nakamura K Inoue H Possible participation of advanced glycation end products in the pathogenesis of colorectal cancer in diabetic patients Med Hypothesis 2005 64 1208 1210 10.1016/j.mehy.2005.01.015 15823719

[B29] Nessar A Advanced glycation end products-role in pathology of diabetic complications Diab Res Clin Pract 2005 67 3 21 10.1016/j.diabres.2004.09.004 15620429

[B30] Matsuura N Aradate T Sasaki C Kojima H Ohara M Hasegawa J Ubukatu M Screening system for the Maillard reaction inhibitor from natural product extracts J Heal Sci 2002 48 520 526 10.1248/jhs.48.520

[B31] Thornalley PJ Use of aminoguanidine (pimagedine) to prevent the formation of advanced glycation endproducts Archiv Biochem Biophys 2003 419 31 40 10.1016/j.abb.2003.08.013 14568006

[B32] Ou P Wolff SP Aminoguanidine: a drug proposed for prophylaxis in diabetes inhibits catalase and generates hydrogen peroxide *in vitro* Biochem Pharmacol 1993 46 1139 1144 10.1016/0006-2952(93)90461-5 8216363

